# Supplementary material for: A targeted LC MS/MS assay of a health surveillance panel and its application to chronic kidney disease
Source: bioRxiv. 2025 Mar 16:2025.03.14.643399. Preprint. [Version 1] doi: 10.1101/2025.03.14.643399 (PMC11952516; doi:10.1101/2025.03.14.643399)
Supplement: Supplement 2 [file media-2.pdf]

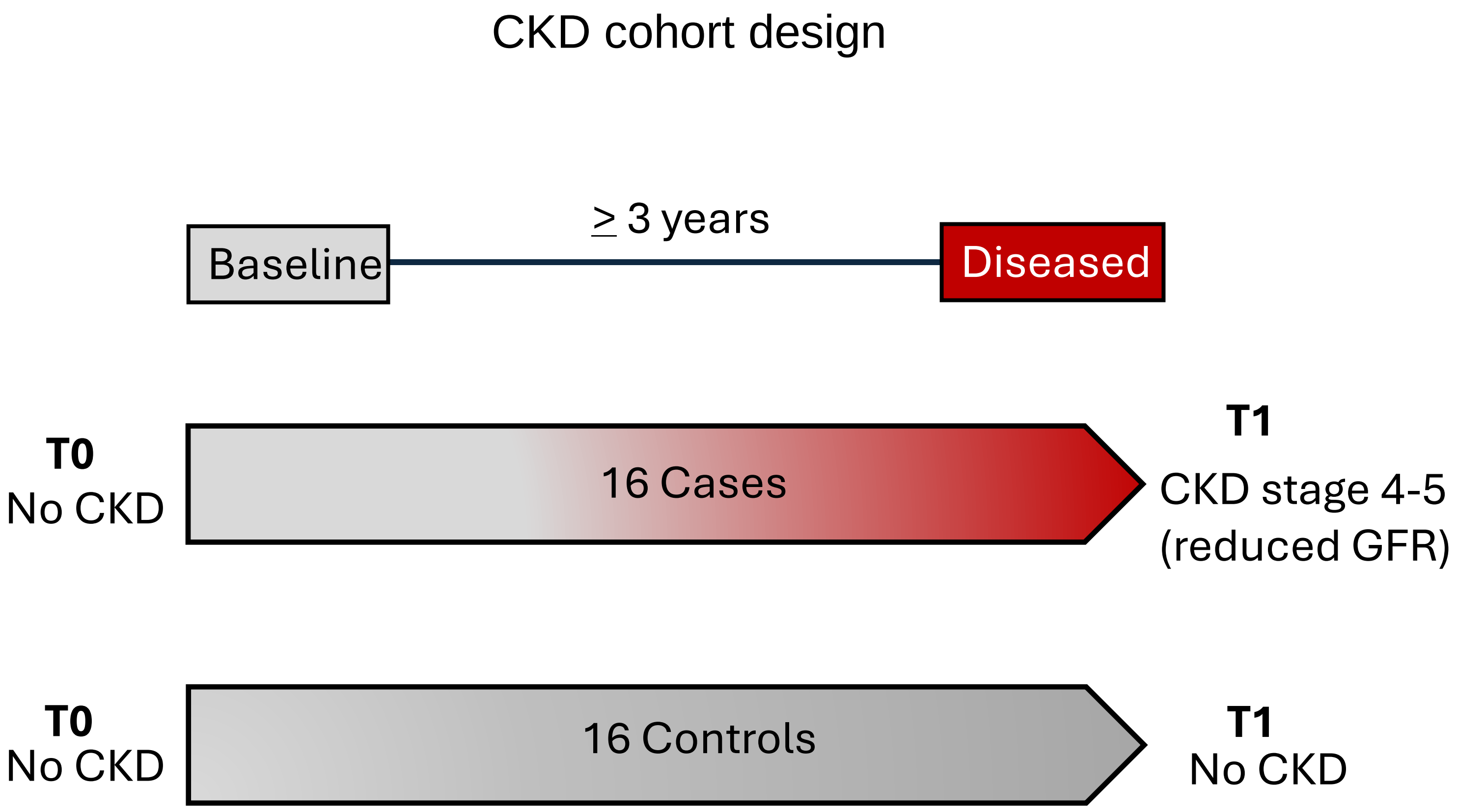

FigureS1. CKD cohort design.

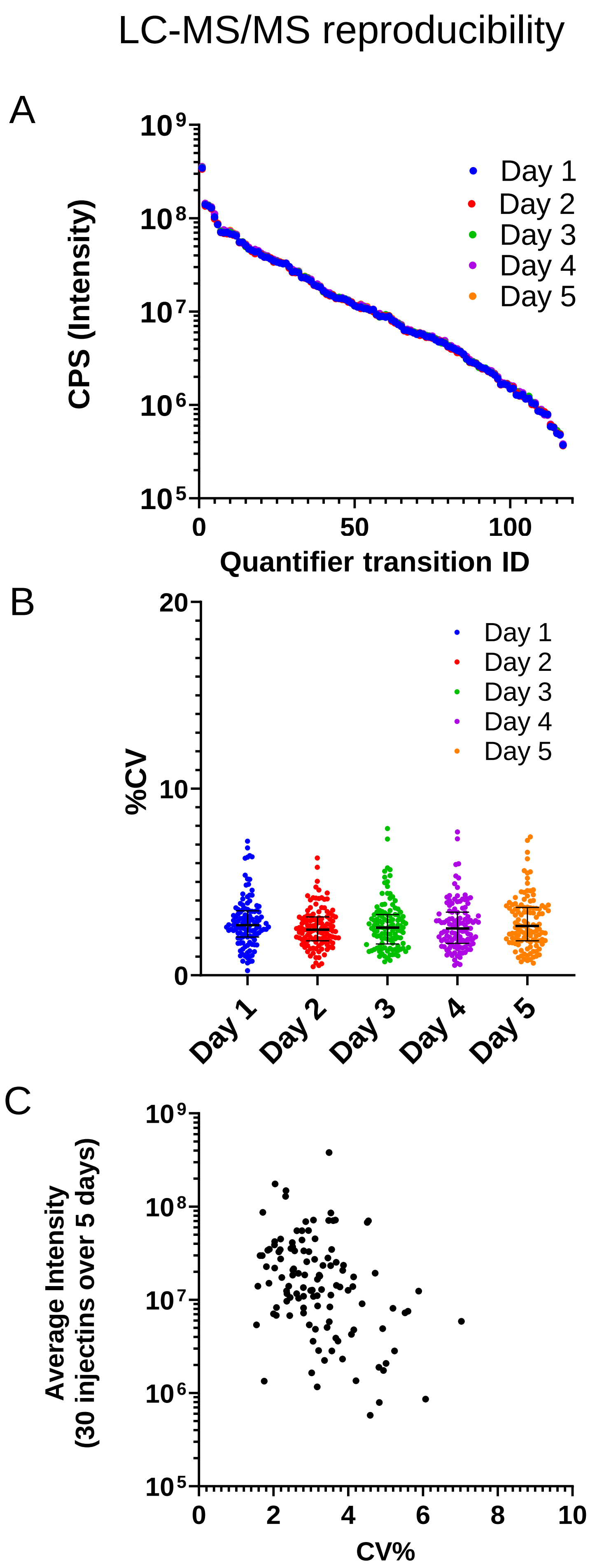

FigureS2. LC-MS/MS reproducibility. SIL peptides in digested control pooled plasma were injected 6 times per day on 5 separate days. A) Average intensities. B) Intra-day %CV. C) Inter-day %CV versus average intensities.

SIL peptide stability on the autosampler at 14°C

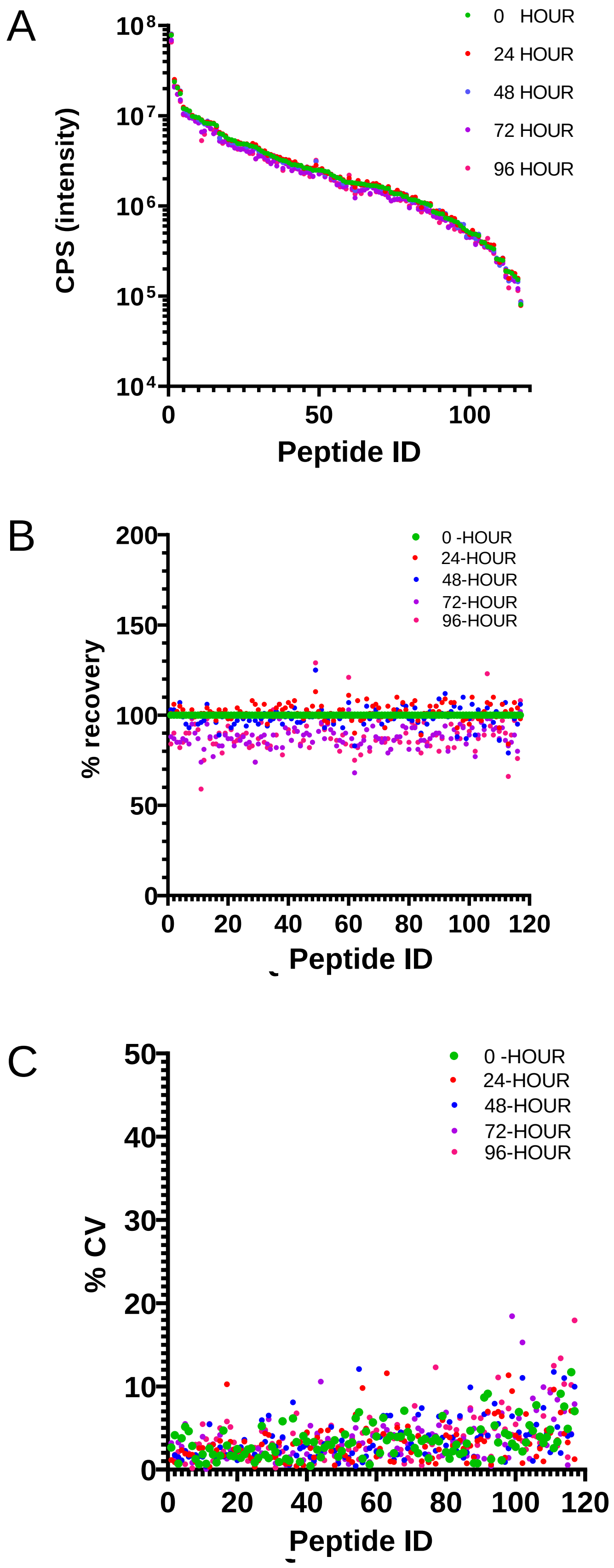

FigureS3. SIL peptides were stored at 14 ºC in the autosampler of the HPLC. At 24-hour intervals, three injections were taken from the same vial to quantify 117 peptides and determine intensity (A), recovery (B), and %CV (C).

Antithrombin-III (ANT3) and Immunoglobulin heavy constant alpha 1 (IGHA1) in CDK

A

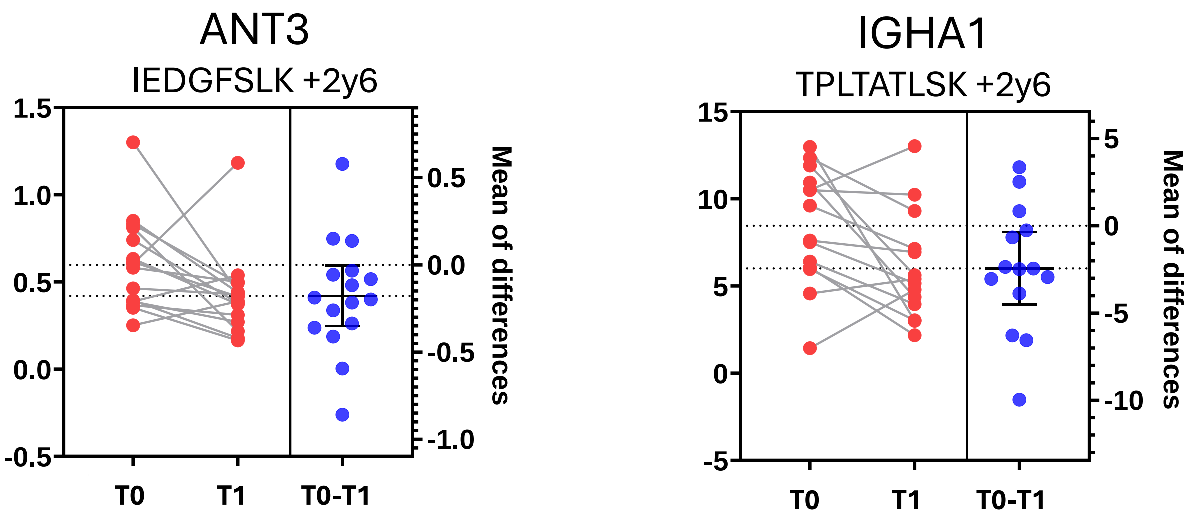

B

Compare Time Points (T0 vs T1)

Protein	Peptide	Ion	Cases (T0 vs T1) P value	Controls (T0 vs T1) P value
ANT3	IEDGFSLK	2y6	***0.045***	0.865
		2y7	***0.033***	0.901
IGHA1	TPLTATLSK	2y6	***0.024***	0.569
		2y7	***0.014***	0.541

Compare Groups (Case vs control)

Protein	Peptide	Ion	Cases vs Controls (T0) P value	Cases vs Controls (T1) P value
ANT3	IEDGFSLK	2y6	*0.062*	0.840
		2y7	*0.057*	0.848
IGHA1	TPLTATLSK	2y6	*0.866*	0.223
		2y7	*0.919*	0.233

FigureS4. Reduced expression of Antithrombin-III (ANT3) and Immunoglobulin heavy constant alpha 1 (IGHA1) correlates with Chronic Kidney Disease (CKD). (A) ANT3 and IGHA1 proteotypic peptides were quantified in samples at the baseline (T0) and final (T1) timepoints. The graphs present means of measurements from diseased individuals (red) and show the change over time with 95% confidence intervals for the mean (blue). (B) Paired T tests reveal a statistically significant reduction in ANT3 and IGHA1 at the T1 timepoint in individuals with CKD.

Quantification of ANT3 and IGHA1

A

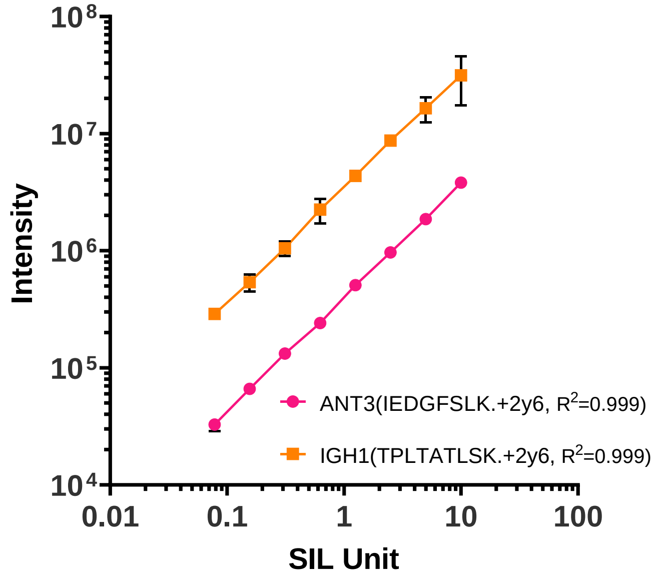

B

Peptide Quantification Characteristics

Protein	Peptide	Ion	Transition type	ULOQ			LLOQ		
				ULOQ (SIL unit)	recovery	CV%	LLOQ (SIL unit)	Recovery (LLOQ)	CV% (LLOQ)
ANT3	IEDGFSLK	+2y6	Quantifier	10	100%	2%	≤ 0.0780	100%	12%
		+2y7	Qualifier	10	91%	4%	≤ 0.0781	91%	12%
IGHA1	TPLTATLSK	+2y6	Quantifier	5	87%	4%	≤ 0.0782	87%	1%
		+2y7	Qualifier	5	90%	5%	≤ 0.0783	90%	6%

Figure 5S. (A) Linearity of SIL peptide quantitation in plasma matrix. (B) Upper and lower limits of quantification of quantifier and qualifier transitions from the SIL peptides.
